# Supplementary material for: Synthesis, Photoisomerization, Antioxidant Activity, and Lipid-Lowering Effect of Ferulic Acid and Feruloyl Amides
Source: Molecules. 2020 Dec 28;26(1):89. doi: 10.3390/molecules26010089 (PMC7794914; doi:10.3390/molecules26010089)
Supplement: Supplementary file 1 [file molecules-26-00089-s001.pdf]

Supplementary Materials

Synthesis, photoisomerization, antioxidant activity and lipid-lowering effect of ferulic acid and feruloyl amides

Chiara Lambruschini, Ilaria Demori, Zeinab El Rashed, Leila Rovegno, Elena Canessa, Katia Cortese, Elena Grasselli, Lisa Moni

Table of contents

- Copies of UV-Vis spectra of t-FEF77 and c-FEF77 S2
- Copies of ^{1}H and ^{13}C NMR spectra of t-FEF77 and c-FEF77 S3

Fig. S1: Copies of UV-Vis spectra of t-FEF77 and c-FEF77 in methanol.

The concentration for UV spectra is 25 μM in MeOH.

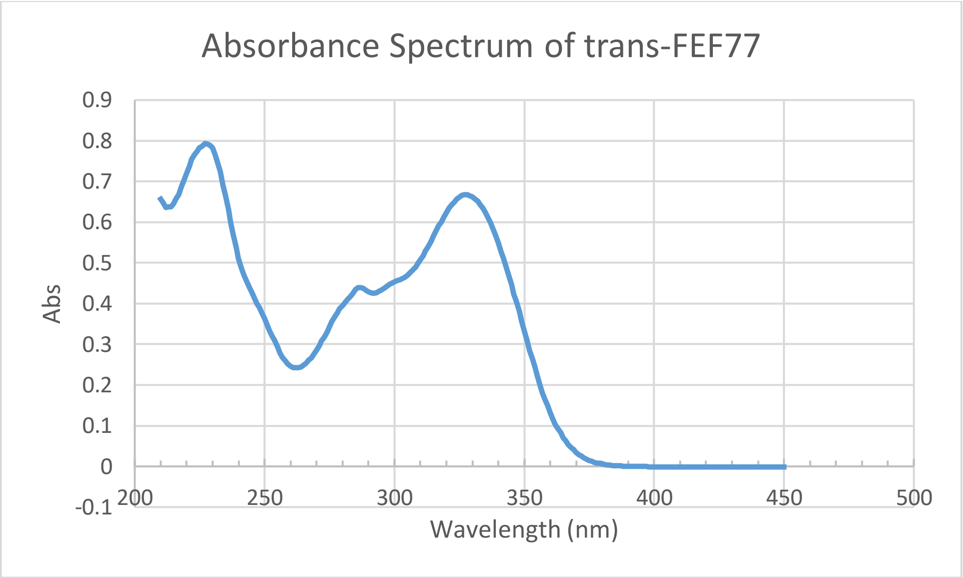

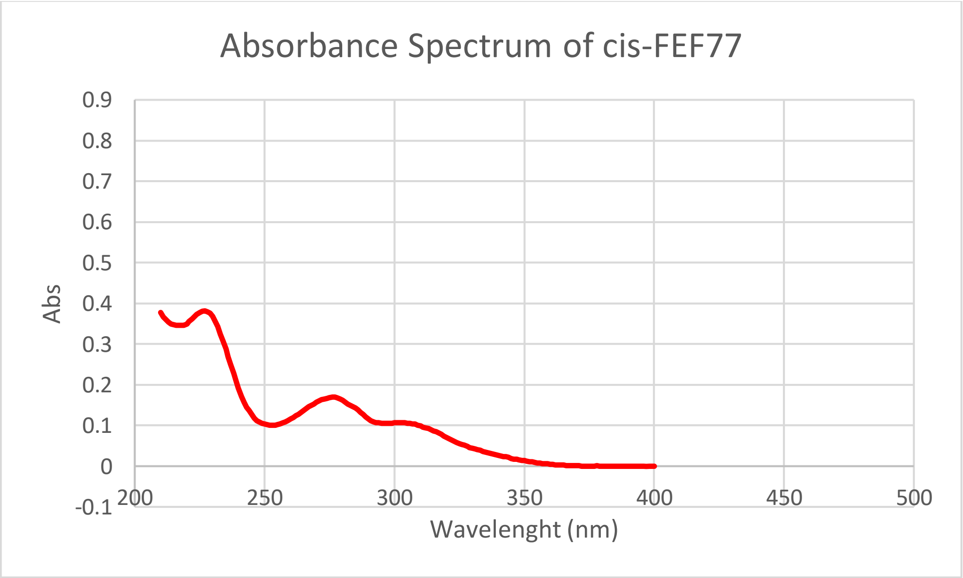

Fig. S2: Copies of ^{1}H and ^{13}C NMR spectra of t-FEF77 and c-FEF77 in CD_3OD

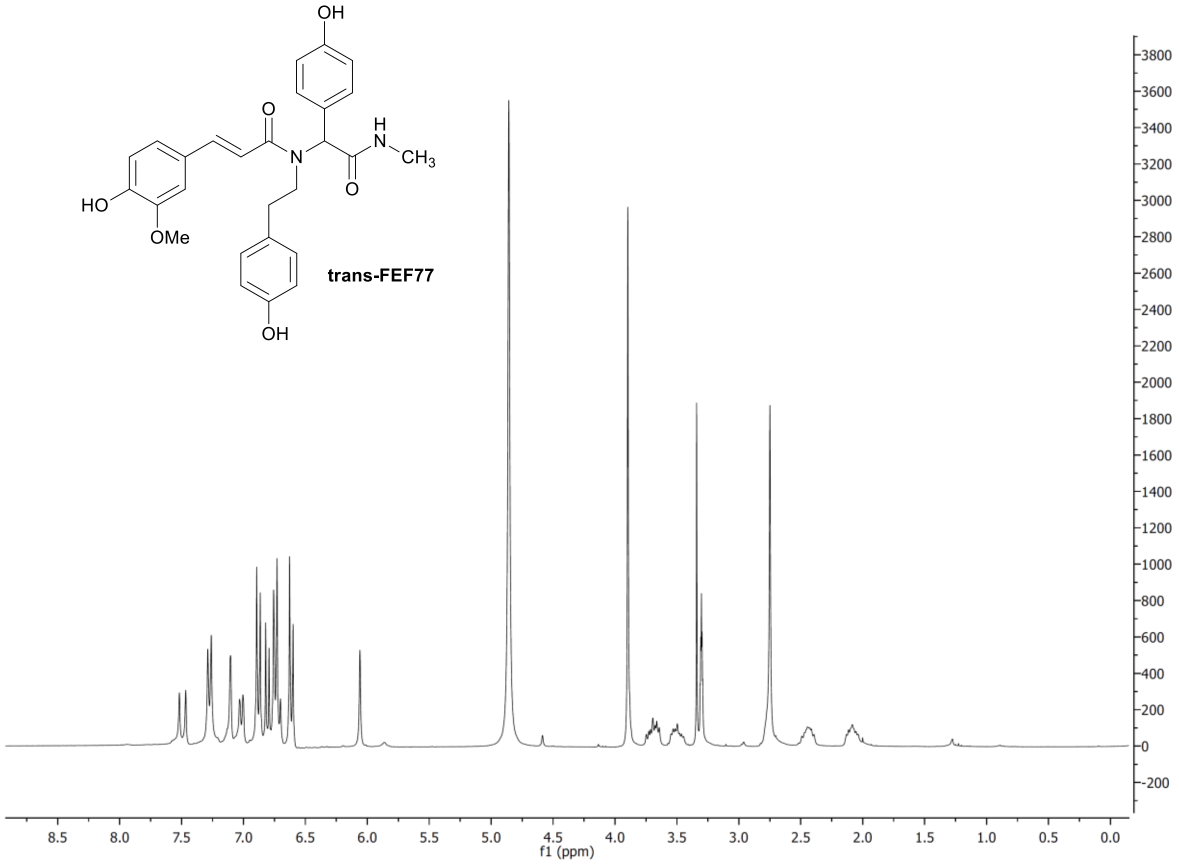

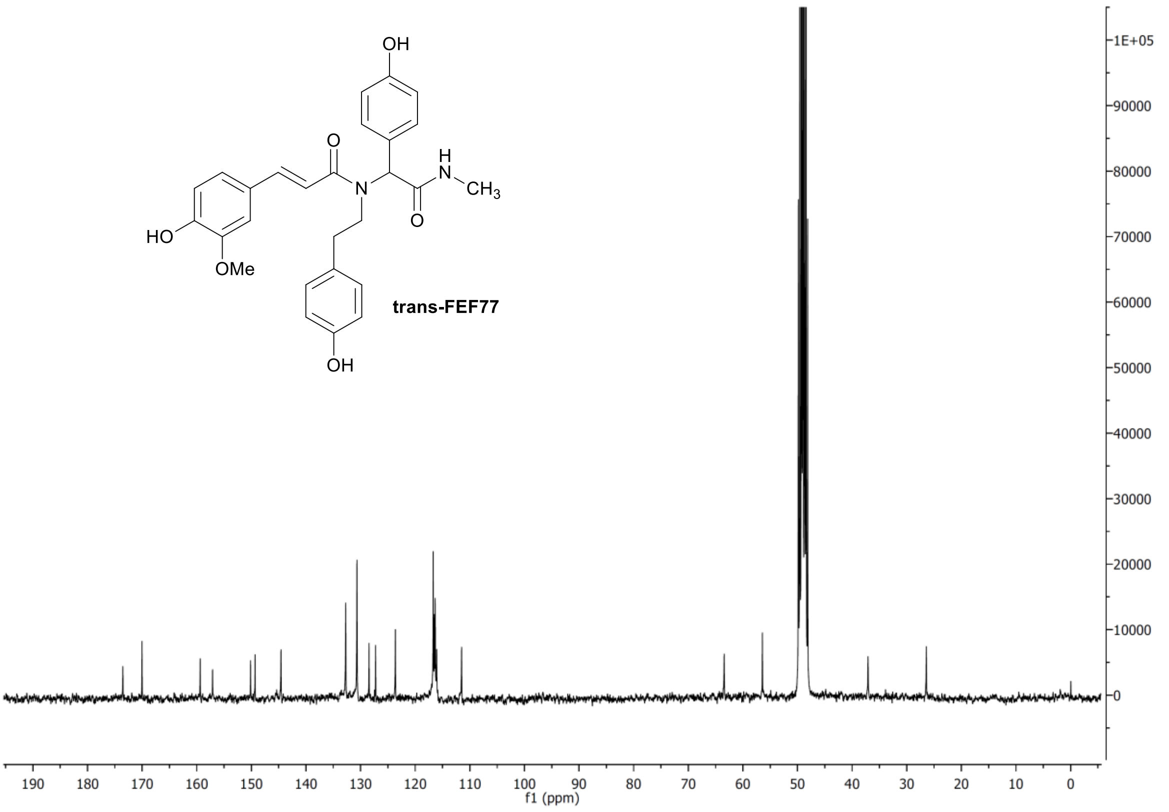

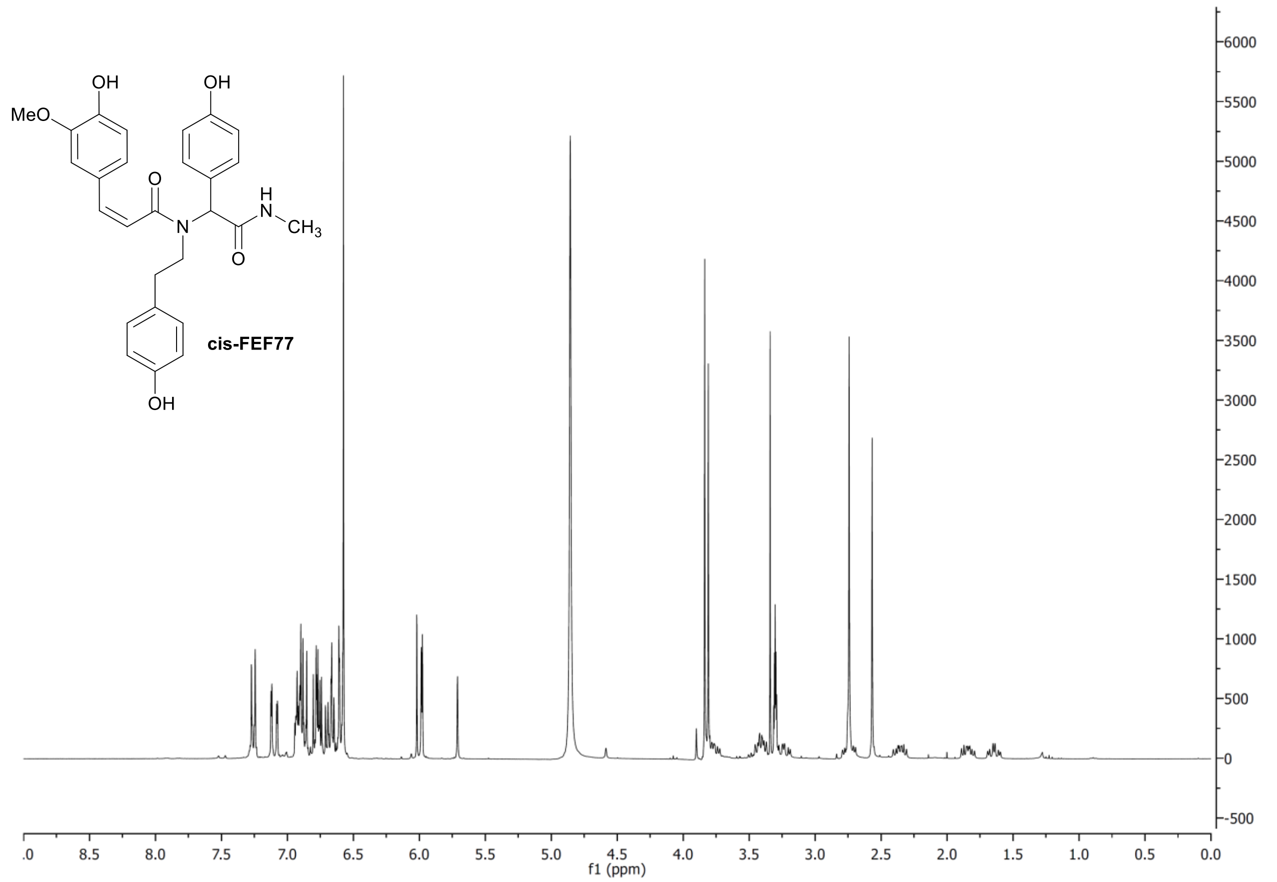

OH
MeO
OH
O
H
N
CH3
N
O
OH
cis-FEF77
f1 (ppm)

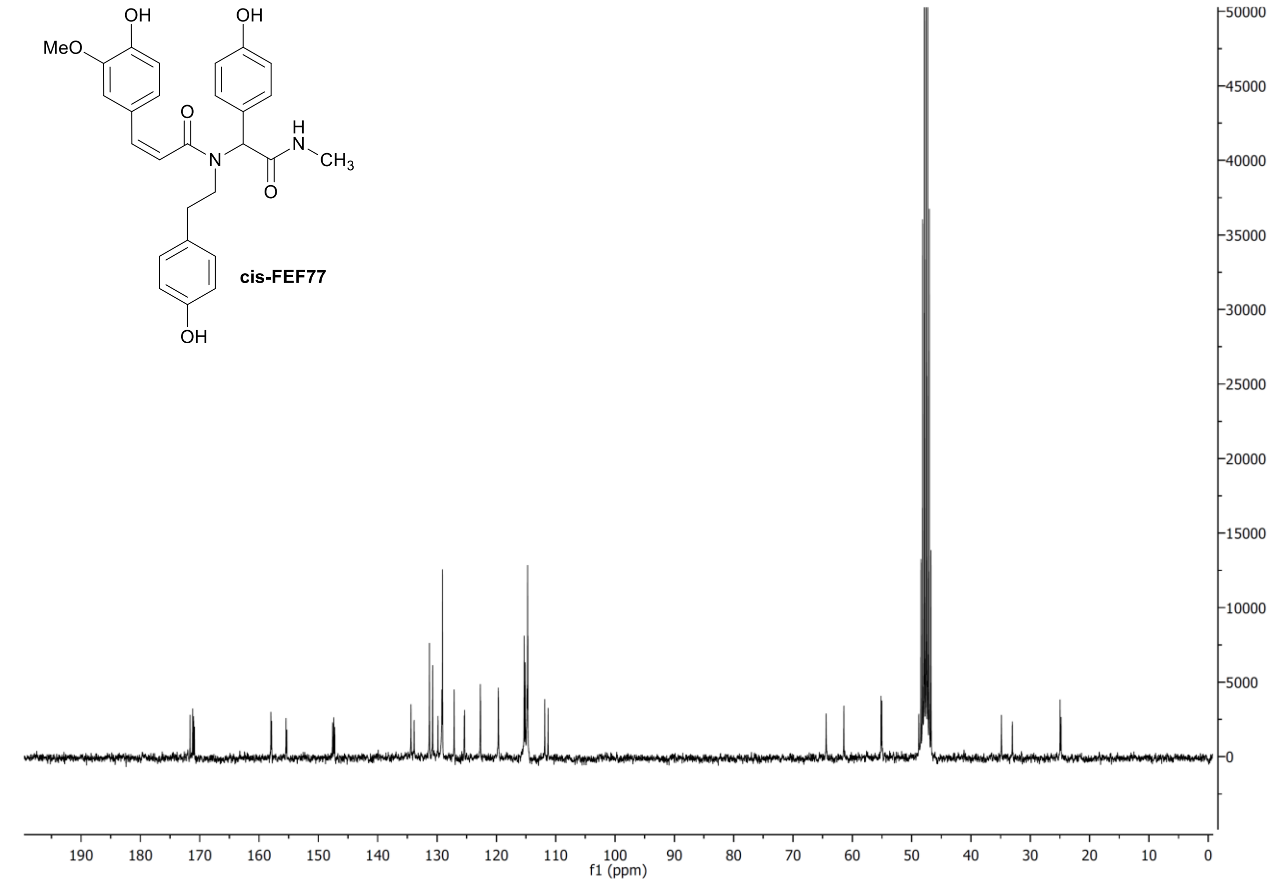

OH
MeO
OH
O
H
N
CH3
N
O
OH
cis-FEF77
f1 (ppm)
